# Single-Cell RNA-seq Analysis Reveals Cellular Functional Heterogeneity in Dermis Between Fibrotic and Regenerative Wound Healing Fates

**DOI:** 10.3389/fimmu.2022.875407

**Published:** 2022-05-17

**Authors:** Cao-Jie Chen, Hiroki Kajita, Kento Takaya, Noriko Aramaki-Hattori, Shigeki Sakai, Toru Asou, Kazuo Kishi

**Affiliations:** ^1^Department of Plastic and Reconstructive Surgery, Keio University School of Medicine, Tokyo, Japan; ^2^Department of Plastic Surgery, Tokyo Cosmetic Surgery Clinic, Tokyo, Japan

**Keywords:** skin wound healing, fibrosis, regeneration, myofibroblast, macrophage, single-cell RNA sequencing

## Abstract

**Background:**

Fibrotic scars are common in both human and mouse skin wounds. However, wound-induced hair neogenesis in the murine wounding models often results in regenerative repair response. Herein, we aimed to uncover cellular functional heterogeneity in dermis between fibrotic and regenerative wound healing fates.

**Methods:**

The expression matrix of single-cell RNA sequencing (scRNA-seq) data of fibrotic and regenerative wound dermal cells was filtered, normalized, and scaled; underwent principal components analysis; and further analyzed by Uniform Manifold Approximation and Projection (UMAP) for dimension reduction with the Seurat package. Cell types were annotated, and cell–cell communications were analyzed. The core cell population myofibroblast was identified and the biological functions of ligand and receptor genes between myofibroblast and macrophage were evaluated. Specific genes between fibrotic and regenerative myofibroblast and macrophage were identified. Temporal dynamics of myofibroblast and macrophage were reconstructed with the Monocle tool.

**Results:**

Across dermal cells, there were six cell types, namely, EN1-negative myofibroblasts, EN1-positive myofibroblasts, hematopoietic cells, macrophages, pericytes, and endothelial cells. Ligand and receptor genes between myofibroblasts and macrophages mainly modulated cell proliferation and migration, tube development, and the TGF-β pathway. Specific genes that were differentially expressed in fibrotic compared to regenerative myofibroblasts or macrophages were separately identified. Specific genes between fibrotic and regenerative myofibroblasts were involved in the mRNA metabolic process and organelle organization. Specific genes between fibrotic and regenerative macrophages participated in regulating immunity and phagocytosis. We then observed the underlying evolution of myofibroblasts or macrophages.

**Conclusion:**

Collectively, our findings reveal that myofibroblasts and macrophages may alter the skin wound healing fate through modulating critical signaling pathways.

## Introduction

The skin is the organ with the largest surface area in the human body that provides an efficient protective barrier against mechanical injury, microbial pathogens, and trauma (1). The skin’s immune system is divided into two structural compartments: epidermis and dermis, both of which contain a plethora of immunocompetent cell types (2). The epidermis is home to the main skin-resident immune cells, Langerhans cells, and melanocytes. Meanwhile, immune-specialized cells like dendritic cells, macrophages, and T cells reside in the dermis (3). The communications within immune populations and the skin environment are critical to the effectiveness of the skin immune system (4). Wound healing is a complex process in the human body, where numerous cell populations with different functions are involved in the stages of hemostasis, inflammatory response, growth, re-epithelialization, and remodeling (5). It is essential to repair the skin after damage (6). Skin wound healing involves three primary phases: inflammation, re-epithelialization, and tissue remodeling (7). Nevertheless, effective therapeutic strategies of accelerating healing and decreasing scarring remain lacking. Single-cell RNA sequencing (scRNA-seq) technology has emerged as an indispensable tool for elucidating cellular phenotype and functional heterogeneity (8). Deciphering the role of each cell type and interactions within cells is of importance to understand the mechanism of normal wound closure (9). Alterations in the microenvironment may influence cellular recruitment or activation, resulting in damaged states of wound healing. ScRNA-seq can be applied for deciphering the cellular changes in chronic wounds and hypertrophic scarring, thereby promoting the development of more effective therapeutic solutions for healing wounds (10). Moreover, in-depth understanding of the differences between fibrotic and regenerative wound healing fates is a prerequisite for developing more effective therapeutic interventions (2). Here, the purpose of this study was to reveal cellular functional heterogeneity in the dermis between fibrotic and regenerative wound healing fates.

## Materials and Methods

### Acquisition of scRNA-seq Profiles

10× genomics scRNA-seq data of regenerative [GSM4213633; large full-thickness excision (1 cm^2^) allows *de novo* follicle regeneration] and fibrotic (GSM4213632; large wounds lead to hairless scars) wound-induced hair neogenesis (WIHN) wounds of adult 6- or 7-week-old C57Bl/6j mice were curated from the Gene Expression Omnibus (GEO) repository (https://www.ncbi.nlm.nih.gov/gds/). The accession number was GSE141814 (11). Regenerative wounds were defined as hair neogenesis, decreased contraction, decreased Wnt and TGF-β signaling activity, and decreased collagen production, while fibrotic wounds were defined as decreased hair neogenesis, increased contraction, increased Wnt and TGF-β signaling activity, and increased collagen production. This dataset was based on the platform of GPL21103 Illumina HiSeq 4000 (*Mus musculus*).

### Quality Control

The DropletUtils package (v 3.13) was adopted to read unique molecular identifiers (UMI) count matrix, identify cells from empty droplets, remove barcode-swapped pseudo-cells, and downsample the count matrix (12). The calculateQCMetrics function of the Scater package was used for counting the expression of genes in cells (13). Cells with proportions of mitochondrial genes ≤ 10% and ribosomal genes ≥ 10% were determined for further analysis.

### Data Preprocessing and Principal Component Analysis

The expression matrix was normalized with the NormalizeData function of the Seurat package (14). The top 2,000 highly variable genes were screened by the FindVariableFeatures function. Then, expression data were linearly scaled utilizing the ScaleData function. Finally, principal component analysis (PCA) was performed with the RunPCA function based on the 2,000 genes.

### Cell Cluster and Annotation

The principal components with large standard deviations were selected. Then, cell clustering analysis was performed using the FindNeighbors and FindClusters function of the Seurat package. With the RunUMAP function, Uniform Manifold Approximation and Projection (UMAP) was carried out for dimension reduction. Cell types were annotated on the basis of the known marker genes.

### Identification of Novel Marker Genes

To calculate the differentially expressed genes between each cluster and all other cells, the FindAllMarkers function of the Seurat package was used and novel marker genes were identified according to the following criteria: |log fold change (FC)| ≥ 0.1, the minimum expression ratio of cell population = 0.25, and *p*-value ≤ 0.05.

### Ligand–Receptor Network Analysis

Based on the ligand–receptor pairs from the previous literature (15), the relationship pairs of receptors and ligands were analyzed based on the marker genes of various cells. Then, a cell–cell communication network was conducted and visualized with the Cytoscape software (16). The core cell population was identified according to the largest number of receptor–ligand pairs in the network. Moreover, the receptor and ligand genes were extracted.

### Function Enrichment Analysis

Function enrichment analysis of the indicated genes was carried out utilizing the clusterProfiler package, including Gene Ontology (GO) and Kyoto Encyclopedia of Genes and Genomes (KEGG) pathway analysis (17). GO categories contain biological process, cellular component, and molecular function. Terms with *p* < 0.05 were considered significantly enriched.

### Protein–Protein Interaction Analysis

The Search Tool for the Retrieval of Interacting Genes (STRING) database (version 11.0; https://string-db.org/) was utilized for exploring the functional interactions between marker gene-encoded proteins (18). Then, PPI networks were constructed and the top 20 hub genes were identified.

### Pseudotime Analysis

Pseudotime analysis was carried out with the Monocle 3 tool (19). Firstly, genes that were expressed in at least 5% of the cells were selected. Then, the reduceDimension function was utilized to perform dimensionality reduction analysis, followed by cell cluster with the clusterCells function. Afterwards, the differentialGeneTest function was adopted to determine candidate genes with differences between the clusters with *p* < 0.05. The dimensionality reduction analysis of the cells was carried out using the DDRTree approach and the reduceDimension function based on the candidate genes. Through the orderCells function, the cells along the quasi-chronological trajectory were sorted and visualized.

### Gene Set Variation Analysis

The single-sample gene set enrichment analysis (ssGSEA) function of the Gene Set Variation Analysis (GSVA) package was utilized for comparisons of the differences in GO and KEGG terms between groups (20).

### Isolation and Culture of Fibroblasts

C57BL/6 male mice (8–10 weeks old; Sankyo) were used for fibroblast isolation. Briefly, mice were sacrificed by cervical dislocation. The trunk skin was separated in the ultra-clean bench, immersed in 75% ethanol for disinfection, and then cut into small pieces. Blood was removed by rinsing with PBS buffer and transferred evenly to cell culture dishes. DMEM complete medium (Wako) was added to submerge the tissue block that was placed in a constant temperature incubator to fully cultivate. After 24 h, DMEM complete medium was added, which was replaced every 3 days. The mouse skin fibroblasts were purified by the differential adhesion method and were used for subsequent experiments. Our study was approved by the Animal Ethics Committee of Keio University School of Medicine [12090(5)].

### Transfection

Using the TransIT-TKO Transfection Reagent (Mirus), siRNA-Engrailed-1 (horizon) and siRNA-control were transfected into fibroblasts in a constant-temperature incubator. Forty-eight hours later, the knockdown effect of siRNA was confirmed by real-time quantitative polymerase-chain reaction (RT-qPCR).

### RT-qPCR

Total RNA was extracted from fibroblasts using the Isogen reagent (Nippon Gene) following the manufacturer’s instructions. cDNA synthesis was achieved based on the cDNA Synthesis System (Bio-Rad). RT-qPCR was carried out utilizing SYBR Qpcr Mix (Toyobo) on a 7500 Real-Time PCR system (Applied Biosystems). The primer sequences were as follows: EN1, 5’-ACACAACCCTGCGATCCTACT-3’(forward) and 5’-GGACGGTCCGAATAGCGTG-3’ (reverse); ACTB, 5’-GGC TGTATTCCCCTCCATCG-3’(forward) and 5’-CCAGTTGGTAACAATGCCATGT-3’ (reverse). The relative expressions were calculated with the 2^−ΔΔCt^ method.

### Wound Healing Assay

Fibroblasts were plated onto a 6-well plate (about 3 × 10^5^ cells/well). When the confluence reached 100%, the fibroblast monolayer was scratched with a 1000-μl pipette tip. Additionally, detached fibroblasts were removed with serum-free medium. At 0 h and 24 h, the wounded area was photographed.

### Statistical Analysis

All statistical analysis was performed using the R language (version 3.6.1) and R Bioconductor packages. *p* < 0.05 indicated statistical significance.

## Results

### Quality Control of scRNA-seq Data of Fibrotic and Regenerative Wound Dermal Cells

Herein, we collected scRNA data of dermal cells from large skin wounds on day 18 with two distinct healing fates (fibrosis: GSM4213632 or regeneration: GSM4213633) from the GSE141814 dataset. Before analysis, we presented quality control of scRNA data. Barcode rank plots separately depicted the distribution of barcodes in total UMI count for fibrotic and regenerative wound dermal cells (**Supplementary Figures 1A, B**). Knee and inflection points in the barcode rank plots indicated the transition of the total UMI count distribution, which reflected the difference between empty droplets and cell droplets. After filtrating empty droplets, we counted the expression of genes in each cell (**Supplementary Figures 1C, D**). Afterwards, we filtrated out cells with proportions of mitochondrial genes > 10% and ribosomal genes < 10% (**Supplementary Figures 1E, F**).

### Cell Cluster of Fibrotic and Regenerative Wound Dermal Cells

**Figure 1:**
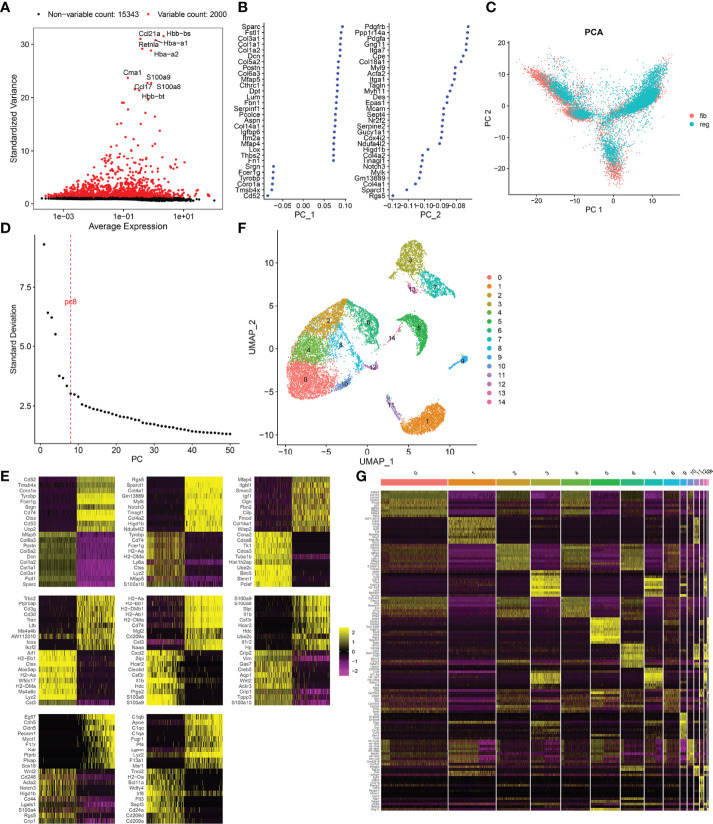
Cell cluster of fibrotic and regenerative wound dermal cells. **(A)** The top 2,000 highly variable genes across fibrotic and regenerative wound dermal cells according to standard deviation. Red dots meant highly variable genes. The top ten highly variable genes were marked. **(B)** Two of the most principal components according to standard deviation. **(C)** PCA plots of wound dermal cells between fibrotic (fib) and regenerative (reg) conditions. Reference atlas was colored by tissue of origin (fibrotic and regenerative wounds). **(D)** Determination of the optimal principal components through elbow plot. **(E)** Heatmaps showing the top 20 marker genes in each principal component. **(F)** Cell cluster based on the screened principal components. **(G)** Heatmap showing the expression patterns of the top ten marker genes in each cell cluster.

After normalizing scRNA data, we screened the top 2,000 highly variable genes across fibrotic and regenerative wound dermal cells (**Figure 1A**). Then, scRNA data were linearly scaled and analyzed by dimensionality reduction with PCA. Here, we screened the top two principal components for subsequent analysis (**Figure 1B**). PCA results uncovered the prominent difference between fibrotic and regenerative wound dermal cells (**Figure 1C**). According to the elbow point, we identified the optimal principal components as 8 (**Figure 1D**). Heatmaps depicted the top 20 marker genes in each principal component (**Figure 1E**). With the UMAP method, dermal cells were clustered into 15 clusters (**Figure 1F**). The top ten marker genes of each cell cluster are presented in **Figure 1G**.

### Identification of Cell Types and Their Marker Genes Across Fibrotic and Regenerative Wound Dermal Cells

**Figure 2:**
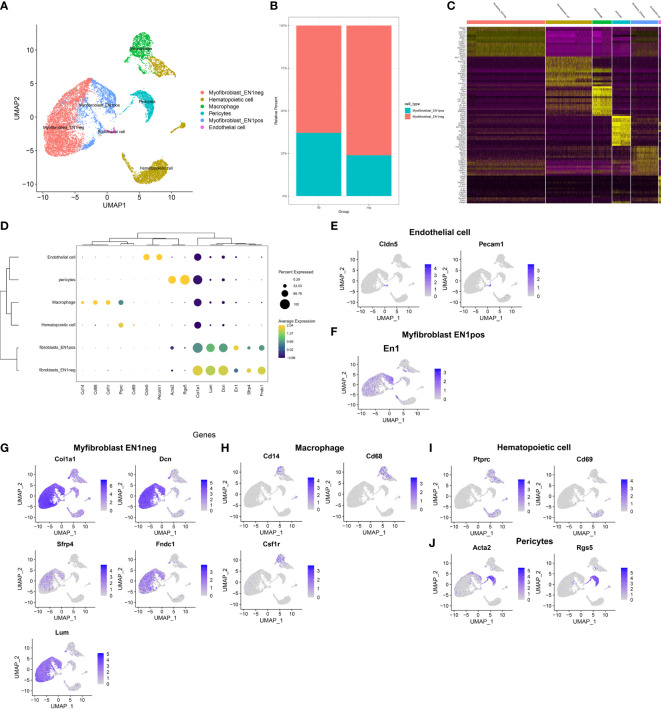
Identification of cell types and their marker genes across fibrotic and regenerative wound dermal cells. **(A)** UMAP plots showing cell types identified by marker genes. Each cell type was colored by a unique color. **(B)** The cell ratio of EN1-negative and -positive myofibroblasts among fibrotic and regenerative wound dermal cells. **(C)** Heatmap visualizing cell-type-specific gene expression patterns. Each column represented the average expression after cells were grouped. **(D)** Integrated analysis showing marker genes across cell types. The size of each circle reflected the percentage of cells in each cell type where the gene was detected, and the color shadow reflected the average expression level within each cell type. **(E–J)** UMAP plots of expression of the marker genes for endothelial cells, EN1-negative and -positive myofibroblasts, macrophages, hematopoietic cells, and pericytes.

This study attempted to identify cell types across fibrotic and regenerative wound dermal cells. Based on the known marker genes, six cell types were annotated, as follows: EN1-negative myofibroblasts (*n* = 6,392), EN1-positive myofibroblasts (*n* = 2,219), hematopoietic cells (*n* = 3,774), macrophages (*n* = 1,461), pericytes (*n* = 1,493), and endothelial cells (*n* = 303; **Figure 2A**). **Table 1** lists the cell ratio of each cell type. In particular, we noticed the differences in ratios of EN1-negative and -positive myofibroblasts between fibrotic and regenerative wound dermal cells (**Figure 2B**). With |logFC| ≥ 0.1, the minimum expression ratio of cell population = 0.25, and *p*-value ≤ 0.05, we identified novel marker genes in each cell type (**Supplementary Table 1**). The top ten marker genes in each cell type were visualized, as follows: EN1-negative myofibroblasts (Aebp1, Col1a1, Col1a2, Col3a1, Col8a1, Dcn, Eln, Mfap2, Mfap4, and Sparc), hematopoietic cells (AW112010, Cd3d, Cd3g, Cd52, Hcst, Ltb, Ptprcap, Rac2, Srgn, and Trbc2), macrophages (Apoe, C1qb, Ccl9, Cd74, Ctss, Fcer1g, H2-Eb1, Lyz2, Ms4a6c, and Tyrobp), pericytes (Acta2, Col4a1, Col4a2, Gm13889, Higd1b, Myl9, Mylk, Rgs5, Sparcl1, and Tagln), EN1-positive myofibroblasts (Birc5, Pclaf, Stnm1, Ube2c, Hist1h2ap, Col5a3, Cks2, Aqp1, Tnfaip6, and Timp1), and endothelia cells (Egfl7, Cldn5, Cdh5, Ramp2, Ecscr, Pecam1, Cd200, Ltbp4, Aqp1, and Hist1h2ap) (**Figure 2C**). Furthermore, we detected the expression levels of the known marker genes that were used for annotating cell types, as follows: endothelial cells (Cldn5, Pecam1, and Cd74), EN1-negative and -positive myofibroblasts (En1, Col1a1, Dcn, Sfrp4, Fndc1, and Lum), macrophages (Cd14, Cd68, and Csf1r), and hematopoietic cells (Ptprc, Cd69, Acta2, and Rgs5) (**Figures 2D–J**).

**Table 1:** Cell ratio of each cell type.

Cell type	Group	Count	Total	Ratio
Endothelial cell	Fibrotic	76	5,130	0.014815
Endothelial cell	Regenerative	112	10,512	0.010654
EN1-negative myofibroblasts	Fibrotic	772	5,130	0.150487
EN1-negative myofibroblasts	Regenerative	5,620	10,512	0.534627
EN1-positive myofibroblasts	Fibrotic	454	5,130	0.088499
EN1-positive myofibroblasts	Regenerative	1,765	10,512	0.167903
Hematopoietic cell	Fibrotic	2,439	5,130	0.475439
Hematopoietic cell	Regenerative	1,335	10,512	0.126998
Macrophage	Fibrotic	725	5,130	0.141326
Macrophage	Regenerative	851	10,512	0.080955
Pericytes	Fibrotic	664	5,130	0.129435
Pericytes	Regenerative	829	10,512	0.078862

### Cell–Cell Interactions Based on Ligand–Receptor Interactions

**Figure 3:**
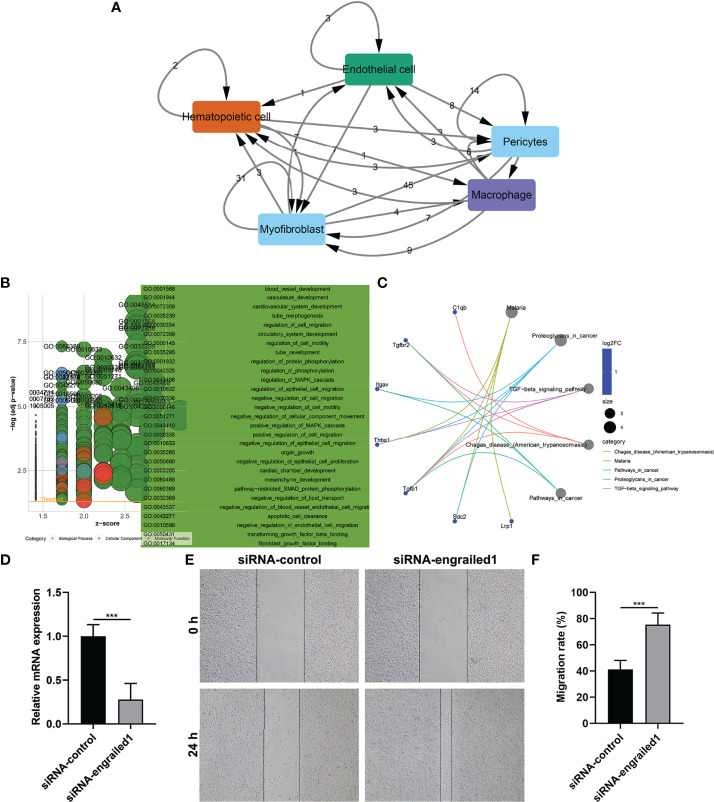
Cell–cell interactions and biological functions of ligand and receptor genes between myofibroblasts and macrophages. **(A)** The network of ligand–receptor-mediated multicellular signaling. The arrow pointed to the recipient cell, and the number on the line indicated the number of receptor–ligand pairs. **(B)** GO enrichment results of ligand and receptor genes between myofibroblasts and macrophages. **(C)** KEGG pathways enriched by ligand and receptor genes between myofibroblasts and macrophages. **(D)** RT-qPCR for the mRNA expressions of EN1 in fibroblasts transfected with siRNA of EN1. **(E, F)** Wound healing assay for the migration of EN1-knockdown fibroblasts. Bar, 20 μm. ***p < 0.001.

Wound healing is a complex process that necessitates the collaborative efforts of diverse cell lineages (21). Cell-to-cell communications across diverse cell types thoroughly govern appropriate functions of metazoans as well as widely rely on interactions between secreted ligands and cell-surface receptors. Based on the marker genes, ligand–receptor interactions were matched. The number of ligands/receptors for myofibroblasts, pericytes, endothelial cells, macrophages, and hematopoietic cells was 114, 91, 32, 28 and 17, respectively (**Figure 3A**). According to the number of intercellular receptor–ligand pairs, we screened out myofibroblasts as the core cell population.

### Biological Functions of Ligand and Receptor Genes Between Myofibroblasts and Macrophages

We further evaluated the biological functions of ligand and receptor genes between myofibroblasts and macrophages. Our results demonstrated that ligand and receptor genes between myofibroblasts and macrophages were mainly involved in tube morphogenesis and development, regulation of cell migration, and motility (**Figure 3B**). Moreover, we found that the TGF-β signaling pathway was markedly enriched by these ligand and receptor genes between myofibroblasts and macrophages (**Figure 3C**).

### Knockdown of EN1 Facilitates Fibroblast Migration

We further verified the effects of EN1 on the migration of fibroblasts. Firstly, siRNA against EN1 was designed and transected into fibroblasts. RT-qPCR demonstrated that EN1 mRNA expression was distinctly reduced following siRNA-EN1 transfection (**Figure 3D**). According to wound healing results, EN1-knockout fibroblasts displayed significantly enhanced migration capacity (**Figures 3E, F**). Hence, EN1 suppression enabled to facilitate fibroblast migration.

### Identification of Specific Genes Between Fibrotic and Regenerative Myofibroblasts and Their Biological Functions

**Figure 4:**
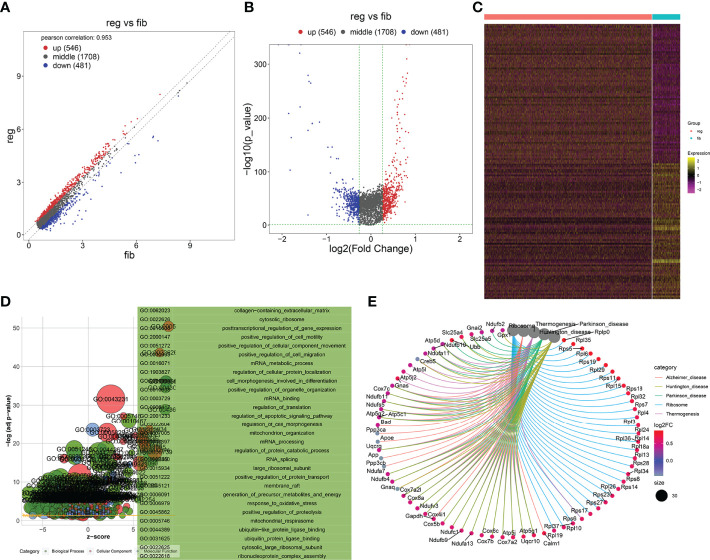
Identification of specific genes between fibrotic and regenerative myofibroblasts and their biological functions. **(A, B)** Scatter plots and volcano diagram for the up- and downregulated specific genes in regenerative (reg) compared to fibrotic (fib) myofibroblasts. Red dots meant upregulated genes while blue dots meant downregulated genes. **(C)** Heatmap visualizing the expression patterns of the specific genes in fibrotic and regenerative myofibroblasts. Yellow represented upregulation and purple represented downregulation. **(D)** GO enrichment results of specific genes that were abnormally expressed between fibrotic and regenerative myofibroblasts. **(E)** KEGG pathways involved in specific genes that were abnormally expressed between fibrotic and regenerative myofibroblasts.

With the cutoffs of |FC| > 1.2 and *p* < 0.05, we identified 546 up- and 481 downregulated specific genes in regenerative compared to fibrotic myofibroblasts (**Figures 4A–C**). **Table 2** lists the first 20 up- and downregulated specific genes between regenerative and fibrotic myofibroblasts. As depicted in **Figure 4D**, we observed that the specific genes markedly participated in collagen-containing extracellular matrix, posttranscriptional regulation of gene expression, positive regulation of cell migration, mRNA metabolic process, and apoptotic signaling pathway. Moreover, ribosome and thermogenesis were prominently enriched by the specific genes (**Figure 4E**).

**Table 2:** The first 20 up- and downregulated specific genes between fibrotic and regenerative myofibroblasts.

Gene	log2FC	*p*-value	*Q*-value	Regenerative	Fibrotic
Rplp0	0.870992	0	0	5.166991	4.295999
Ifitm2	0.843781	1.12E−173	1.94E−169	3.837826	2.994046
Mfap5	0.826158	5.93E−128	1.03E−123	4.591184	3.765026
Lgals1	0.820706	4.86E−284	8.43E−280	6.19352	5.372813
Hist1h2bc	0.81979	4.50E−90	7.81E−86	2.042755	1.222965
Serf2	0.805752	1.37E−310	2.39E−306	4.973459	4.167707
Rpl35	0.801322	0	0	5.164454	4.363133
Rps5	0.795055	5.07E−274	8.79E−270	4.725084	3.930029
Basp1	0.794315	1.55E−93	2.69E−89	2.268422	1.474106
Rpl6	0.792999	4.84E−266	8.40E−262	4.489802	3.696803
Ybx1	0.791379	6.39E−117	1.11E−112	2.98192	2.19054
Rps19	0.790084	0	0	5.198609	4.408525
Ost4	0.782118	2.55E−123	4.42E−119	3.079057	2.296939
Rpl29	0.780779	1.14E−175	1.98E−171	3.875578	3.094799
H19	0.767949	8.58E−45	1.49E−40	3.185378	2.417429
Rps11	0.763653	3.10E−260	5.37E−256	4.655295	3.891641
Rpl15	0.760256	2.28E−207	3.96E−203	4.262648	3.502392
Ift20	0.758	1.47E−93	2.55E−89	2.397842	1.639842
Ssr4	0.745387	2.11E−101	3.67E−97	2.89302	2.147633
Ubb	0.744921	1.14E−144	1.97E−140	4.529784	3.784862
mt-Nd4l	−2.08112	0	0	0.883721	2.964844
mt-Atp6	−1.85976	0	0	5.349053	7.20881
Hspa1b	−1.85125	4.49E−209	7.79E−205	0.611879	2.463132
mt-Co2	−1.84169	0	0	4.106449	5.948142
AC160336.1	−1.81875	4.98E−104	8.63E−100	0.763221	2.58197
Hspa1a	−1.79337	2.08E−164	3.61E−160	1.385872	3.179244
mt-Nd4	−1.60147	3.51E−321	6.08E−317	3.543676	5.145146
mt-Nd5	−1.59322	2.78E−221	4.83E−217	1.144946	2.738165
mt-Cytb	−1.57454	0	0	4.565919	6.140456
Igfbp2	−1.4162	1.28E−20	2.21E−16	2.045862	3.462061
mt-Nd3	−1.41514	1.13E−177	1.96E−173	1.403288	2.818428
mt-Nd1	−1.4142	4.61E−280	8.00E−276	4.509633	5.923829
mt-Co3	−1.39259	1.24E−268	2.15E−264	5.529273	6.921861
mt-Co1	−1.35374	1.30E−265	2.26E−261	5.598606	6.952347
mt-Nd2	−1.32088	1.81E−190	3.14E−186	2.765453	4.086338
Gm26917	−1.31863	7.03E−191	1.22E−186	0.653702	1.972335
Cd74	−1.15624	2.79E−193	4.84E−189	0.624805	1.781046
Lars2	−0.96874	2.21E−146	3.83E−142	0.232192	1.200933
Luc7l2	−0.91132	1.16E−98	2.01E−94	1.18695	2.098275
Hspg2	−0.90368	3.60E−128	6.24E−124	2.381196	3.284878

### Identification of Specific Genes Between Fibrotic and Regenerative Macrophages and Their Biological Functions

**Figure 5:**
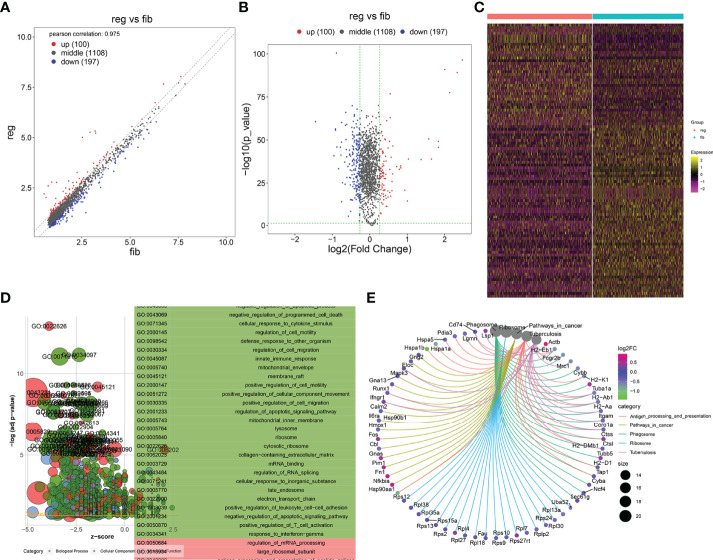
Identification of specific genes between fibrotic and regenerative macrophages and their biological functions. **(A, B)** Scatter plots and volcano diagram showing the up- and downregulated specific genes in regenerative (reg) compared to fibrotic (fib) macrophages. Red dots meant upregulated genes while blue dots meant downregulated genes. **(C)** Heatmap visualizing the expression patterns of the specific genes in fibrotic and regenerative macrophages. Yellow represented upregulation and purple represented downregulation. **(D)** GO enrichment results of specific genes that were abnormally expressed between fibrotic and regenerative macrophages. **(E)** KEGG pathways involved in specific genes that were abnormally expressed between fibrotic and regenerative macrophages.

With the cutoffs of |FC| > 1.2 and *p* < 0.05, we found that 100 specific genes were significantly upregulated while 197 specific genes were significantly downregulated in regenerative compared to fibrotic macrophages (**Figures 5A–C**). **Table 3** lists the first 20 up- and downregulated specific genes between fibrotic and regenerative macrophages. GO enrichment analysis uncovered that the specific genes were markedly involved in the negative regulation of programmed cell death, the regulation of cell migration, innate immune response and apoptotic signaling pathway, collagen-containing extracellular matrix, the positive regulation of T cell activation, and response to interferon γ (**Figure 5D**). Moreover, we observed that antigen processing and presentation, pathways in cancer, phagosome, ribosome, and tuberculosis were prominently enriched by the specific genes (**Figure 5E**).

**Table 3:** The first 20 up- and downregulated specific genes between fibrotic and regenerative macrophages.

Gene name	log2FC	*p*-value	*Q*-value	Regenerative	Fibrotic
Sparc	2.474022	3.60E−97	6.24E−93	5.010571	2.536548
Col1a1	2.33817	6.49E−90	1.13E−85	5.266303	2.928133
Col1a2	2.13485	3.01E−78	5.21E−74	5.327119	3.192269
Col3a1	2.005563	1.16E−91	2.01E−87	5.223726	3.218163
Dcn	1.836106	2.30E−46	3.98E−42	2.785851	0.949745
Bgn	1.83586	5.99E−50	1.04E−45	2.600128	0.764269
Fstl1	1.648779	1.28E−39	2.22E−35	2.200177	0.551399
Postn	1.572566	2.54E−51	4.40E−47	2.775437	1.202871
Mfap5	1.370976	2.18E−39	3.79E−35	2.023966	0.65299
Hbb-bs	1.031846	1.21E−39	2.10E−35	2.844128	1.812282
Cxcl2	1.004274	2.60E−15	4.51E−11	3.268016	2.263742
Actb	0.934603	1.46E−21	2.53E−17	7.663418	6.728815
Klf2	0.828223	1.34E−34	2.33E−30	2.497856	1.669632
Timp2	0.824526	1.09E−35	1.89E−31	1.978589	1.154062
Neat1	0.789153	1.13E−33	1.96E−29	2.328203	1.53905
Nfkbia	0.718421	2.88E−35	4.99E−31	2.761737	2.043317
Lgals1	0.61418	3.23E−47	5.60E−43	4.783109	4.168928
Fn1	0.610899	5.21E−31	9.03E−27	3.726565	3.115666
Pim1	0.59329	1.34E−26	2.32E−22	2.966403	2.373113
Cd63	0.592092	2.84E−21	4.92E−17	2.447508	1.855417
Hspa1b	−1.44863	2.08E−61	3.60E−57	1.266466	2.715092
Hsp90aa1	−0.957	1.59E−41	2.76E−37	2.518111	3.475109
Gm26917	−0.91834	3.81E−57	6.61E−53	0.782974	1.701314
Gm42418	−0.91626	1.85E−56	3.20E−52	1.082872	1.999131
Tpt1	−0.89005	3.21E−101	5.57E−97	4.517284	5.40733
mt-Nd5	−0.87923	1.13E−46	1.96E−42	0.858755	1.737986
Hspa1a	−0.83491	4.80E−34	8.32E−30	3.320621	4.155527
mt-Co2	−0.78506	1.59E−46	2.76E−42	3.967573	4.752638
mt-Atp6	−0.77046	5.82E−42	1.01E−37	4.934988	5.70545
Mycbp2	−0.75645	1.65E−49	2.86E−45	0.967289	1.723739
H2-Eb1	−0.75235	6.73E−15	1.17E−10	5.220528	5.972878
Fcgr2b	−0.75221	7.44E−61	1.29E−56	1.801335	2.553547
Mrc1	−0.72837	6.62E−26	1.15E−21	1.012111	1.740482
mt-Nd4l	−0.67023	7.15E−38	1.24E−33	0.682842	1.35307
AC160336.1	−0.65981	5.00E−25	8.66E−21	1.805651	2.465465
Prkcd	−0.6507	2.95E−59	5.12E−55	1.387319	2.038016
Cybb	−0.64225	8.79E−67	1.52E−62	1.99459	2.636836
Tgfbi	−0.63629	6.10E−51	1.06E−46	2.746255	3.382547
H2-K1	−0.62809	3.72E−45	6.44E−41	2.787025	3.415118
Ier5	−0.61724	5.52E−41	9.58E−37	2.037704	2.654947

### PPI Network Analysis of Specific Genes Between Fibrotic and Regenerative Myofibroblasts or Macrophages

**Figure 6:**
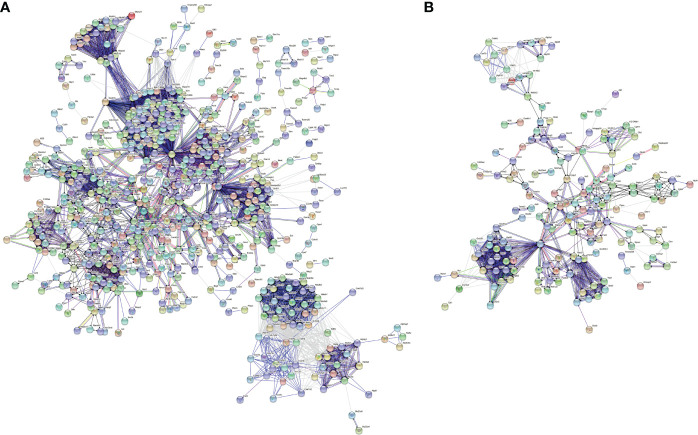
PPI network analysis of specific genes between fibrotic and regenerative myofibroblasts or macrophages. **(A)** The PPI network of specific genes between fibrotic and regenerative myofibroblasts. **(B)** The PPI network of specific genes between fibrotic and regenerative macrophages.

With the STRING tool, we probed the interactions between myofibroblast- or macrophage-specific gene-encoded proteins. In **Figure 6A**, there were 616 nodes in the PPI network of myofibroblasts, reflecting the close interactions of myofibroblast-specific gene-encoded proteins. According to degree, the top 20 nodes were identified as hub genes, including Rps27a, Rps11, Rps23, Rps3, Rps5, Rps15a, Rps6, Rps9, Rps13, Rps14, Rps25, Rps3a1, Rps27, Rps8, Rps19, Rps28, Rps7, Rpl8, Rps18, Rpl26, Rpl32, and Rps16, indicating that the above genes were the core of the network. **Figure 6B** depicts the interactions between macrophage-specific gene-encoded proteins. The 20 hub genes were as follows: Uba52, Rps9, Gnb2l1, Rpl27, Rpl38, Rps13, Rps15a, Fau, Rpl18, Rpl30, Rpl35a, Rpl7, Rplp2, Rps24, Rpl13a, Rpl4, Rps10, Rps12, Rps27rt, and Rps2. The above genes deserve in-depth explorations.

### Reconstruction of the Temporal Dynamics of Myofibroblast and Macrophage

**Figure 7:**
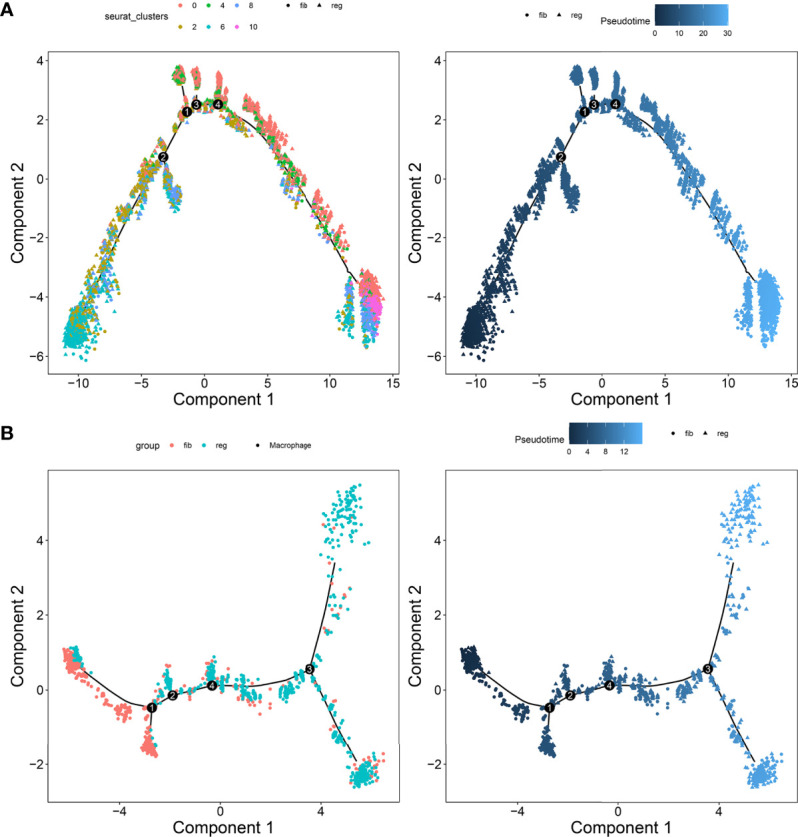
Pseudotime ordering of myofibroblasts and macrophages. **(A)** Myofibroblasts and **(B)** macrophages. Each dot represented one cell and each branch represented one cell state. The left plot was labeled with cell states and the right plot was labeled with developmental time.

To investigate the underlying evolution among myofibroblasts and macrophages, this study adopted the Monocle tool to reveal a pseudotemporal ordering for the similarity of cell clusters with developmental lineages. For myofibroblasts, the results clearly demonstrated the uniform development of myofibroblasts from cluster 6 to cluster 10 (**Figure 7A**). The trends of pseudotime‐dependent genes along the pseudo‐timeline were divided into six cell clusters of myofibroblasts with diverse expression dynamics. Furthermore, we observed that macrophage under fibrotic conditions was in the beginning position of the differentiation process and was sequentially transformed into macrophage under regenerative conditions (**Figure 7B**).

### GSVA Between Clusters 6 and 10 of Fibrotic and Regenerative Myofibroblasts

**Figure 8:**
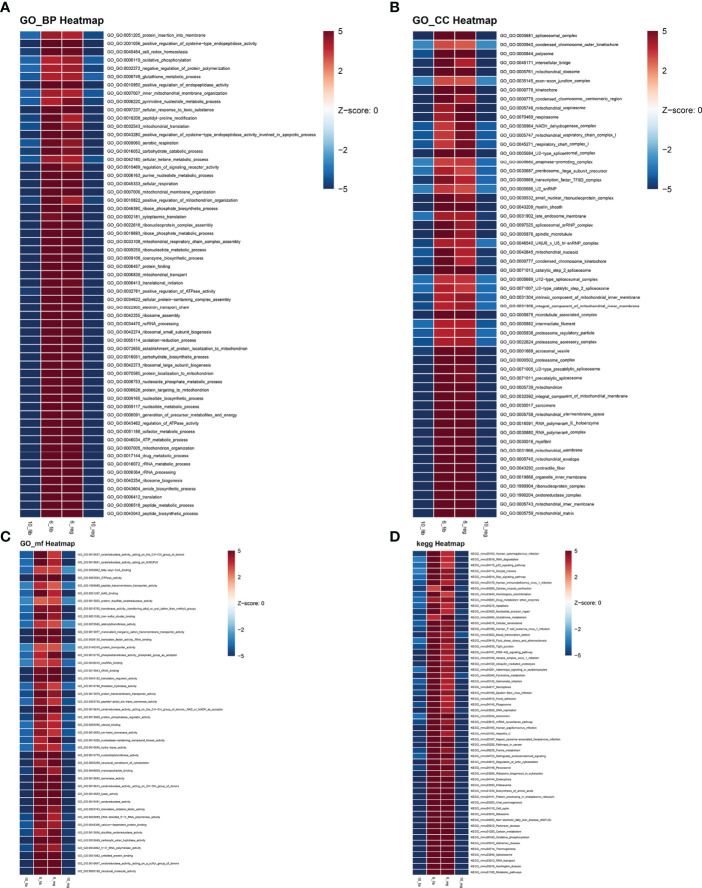
GSVA between clusters 6 and 10 of fibrotic and regenerative myofibroblasts. **(A–D)** Heatmaps showing the differences in activation of biological processes, cellular components, molecular functions, and KEGG pathways between clusters 6 and 10 of fibrotic (fib) and regenerative (reg) myofibroblasts.

According to the results of pseudotime analysis of myofibroblasts, we carried out GSVA between the initially differentiated cluster 6 and the final differentiated cluster 10. Compared with cluster 10 of myofibroblasts in fibrotic and regenerative dermal cells, biological processes such as the metabolic process significantly activated cluster 6 of myofibroblasts in fibrotic and regenerative dermal cells (**Figure 8A**). As depicted in **Figure 8B**, we noticed the prominent activation of cellular components such as mitochondria in cluster 6 of fibrotic and regenerative myofibroblasts in comparison to those in cluster 10. Moreover, we observed that fibrotic and regenerative myofibroblasts in cluster 6 had significantly activated molecular functions like oxidoreductase activity compared with fibrotic and regenerative myofibroblasts in cluster 10 (**Figure 8C**). We also compared the differences in KEGG pathways between clusters. Diverse signaling pathways like metabolic pathways, RNA transport, spliceosome, thermogenesis, oxidative phosphorylation, carbon metabolism, ribosome, cell cycle, protein processing in the endoplasmic reticulum, and biosynthesis of amino acids were prominently activated in fibrotic and regenerative myofibroblasts in cluster 6 compared to those in cluster 10 (**Figure 8D**).

### GSVA Between Fibrotic and Regenerative Macrophages

**Figure 9:**
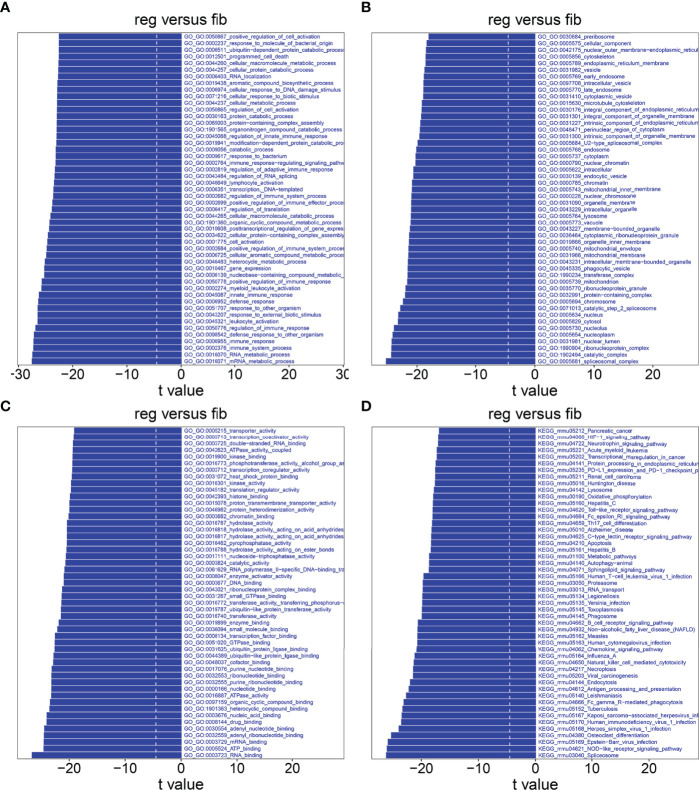
GSVA between fibrotic and regenerative macrophages. **(A–D)** Heatmaps visualizing the differences in activation of biological processes, cellular components, molecular functions, and KEGG pathways between fibrotic (fib) and regenerative (reg) macrophages.

GSVA was also presented between fibrotic and regenerative macrophages. In **Figure 9A**, we determined that biological processes such as the metabolic process and immune response were markedly activated in fibrotic macrophages compared to regenerative macrophages. The significantly activated cellular components such as the spliceosomal complex, catalytic complex, ribonucleoprotein complex, nuclear lumen, nucleoplasm, nucleolus, cytosol, nucleus, catalytic step 2 spliceosome, chromosome, and protein-containing complex were found in fibrotic macrophages compared with regenerative macrophages (**Figure 9B**). As shown in **Figure 9C**, we investigated the marked activation of molecular functions like RNA binding, ATP binding, mRNA binding, adenyl ribonucleotide binding, adenyl nucleotide binding, drug binding, nucleic acid binding, heterocyclic compound binding, organic cyclic compound binding, and ATPase activity in fibrotic macrophages in comparison to regenerative macrophages. Moreover, our results showed that KEGG pathways such as spliceosome, NOD-like receptor signaling pathway, Fc gamma R-mediated phagocytosis, antigen processing and presentation, endocytosis, necroptosis, and natural killer cell-mediated cytotoxicity displayed marked activation in fibrotic macrophages compared to regenerative macrophages (**Figure 9D**).

## Discussion

Skin wound healing involves complicated coordinated interactions within cells. Through scRNA-seq data, this study identified six cell populations, namely, EN1-negative myofibroblasts, EN1-positive myofibroblasts, hematopoietic cells, macrophages, pericytes, and endothelial cells, across the dermis. Evidence suggests that EN1-positive fibroblasts are known to function in scarring, and EN1-negative fibroblasts yield wound regeneration. Thus, we used EN1 as a marker to divide the subgroups. Dynamic cellular events after skin injury rely on bidirectional cell–cell communications against effective wound healing (22). Our results demonstrated the cross-talks between myofibroblasts, hematopoietic cells, macrophages, pericytes, and endothelial cells in the dermis based on the ligand–receptor interactions. As per previous studies, CX3CR1 may mediate the recruitment of bone marrow-derived monocytes or macrophages in skin wound healing, thereby releasing profibrotic as well as angiogenic mediators (23). Moreover, macrophages support proliferation and heterogeneity of myofibroblasts in skin repair (24). Serum endothelial cell-derived extracellular vesicles facilitate diabetic wound healing *via* enhancing myofibroblast proliferation and decreasing senescence (25). Intradermal adipocytes modulate the recruitment of myofibroblasts in skin wound healing (26). Fibroblasts promote NG^2+^ pericyte populations in murine skin development as well as repair (27). On the basis of the above lines of evidence, there were remarkable interplays between diverse cell types during dermis progression. According to the number of ligands and receptors, we identified myofibroblasts as the core cell population. Our function enrichment analyses uncovered that the ligand and receptor genes between myofibroblasts and macrophages were mainly involved in regulating cell proliferation and migration, tube development, and the TGF-β pathway. The TGF-β signaling pathway plays an important role in the formation of collagen in fibroblasts and myofibroblasts (28). Cytokine TGF-β may induce dermal dendritic cells to express IL-31, thereby activating sensory neurons as well as stimulating wound itching during skin would healing (29). Hence, targeting the TGF-β pathway is the promising therapeutic intervention to reduce abnormal skin scar formation.

To explore the differences in molecular mechanisms involving myofibroblasts between fibrotic and regenerative wound healing fates, we identified 546 up- and 481 downregulated specific genes in regenerative compared to fibrotic myofibroblasts. This revealed the heterogeneity of myofibroblasts between fibrotic and regenerative wound healing. Our GO and KEGG enrichment analysis uncovered the key biological functions involving the specific genes between fibrotic and regenerative myofibroblasts. As a result, these specific genes between fibrotic and regenerative myofibroblasts prominently participated in the mRNA metabolic process and organelle organization. Extracellular matrix of connective tissues is synthesized by myofibroblasts that play a critical role in sustaining the structural integrity of various tissues (30).

Skin wound macrophage is an important regulator of skin repair, and its dysfunction may cause chronic and non-healing skin wounds (31). Further analysis identified that 100 specific genes were significantly upregulated while 197 specific genes were significantly downregulated in regenerative compared to fibrotic macrophages. Functional enrichment analysis uncovered that these specific genes between fibrotic and regenerative macrophages primarily participated in regulating inflammatory response, immunity, and phagocytosis. Immunity is the most important function of the skin, which can prevent harmful exposure from the external and internal environment (32). Furthermore, late wound macrophage phagocytosis of the Wnt inhibitor may induce chronic Wnt activity during fibrotic skin healing (11). Collectively, our findings revealed that the heterogeneity of myofibroblasts or macrophages might determine wound healing fate as regenerative or fibrotic.

## Conclusion

Taken together, this study uncovered cellular functional heterogeneity in dermis between fibrotic and regenerative wound healing fates. Moreover, myofibroblasts and macrophages may change the skin wound healing fates by modulating critical signaling pathways. Therefore, our data provided an insight into the development of more effective therapeutic interventions for improving healing fates.

## Data Availability Statement

The datasets presented in this study can be found in online repositories. The names of the repository/repositories and accession number(s) can be found at: https://www.ncbi.nlm.nih.gov/, GSM4213633; https://www.ncbi.nlm.nih.gov/, GSM4213632; https://www.ncbi.nlm.nih.gov/, GSE141814.

## Ethics Statement

Ethical review and approval were not required for the study on human participants in accordance with the local legislation and institutional requirements. Written informed consent for participation was not required for this study in accordance with the national legislation and the institutional requirements. The animal study was reviewed and approved by Keio University School of Medicine. Written informed consent was not obtained from the individual(s) for the publication of any potentially identifiable images or data included in this article.

## Author Contributions

C-JC, HK, and KT: conception or design of the work. C-JC, HK, KT, NA-H, SS, TA, and KK: acquisition, analysis, or interpretation of data. C-JC, HK, KT, NA-H, SS, TA, and KK: drafting the manuscript or revising it critically for important intellectual content. All authors contributed to the article and approved the submitted version.

## Funding

This work was supported in part by Japan China Sasakawa Medical Fellowship (2017816).

## Conflict of Interest

The authors declare that the research was conducted in the absence of any commercial or financial relationships that could be construed as a potential conflict of interest.

## Publisher’s Note

All claims expressed in this article are solely those of the authors and do not necessarily represent those of their affiliated organizations, or those of the publisher, the editors and the reviewers. Any product that may be evaluated in this article, or claim that may be made by its manufacturer, is not guaranteed or endorsed by the publisher.

## Supplementary Material

Supplementary Figure 1 Quality control of scRNA-seq data of fibrotic and regenerative wound dermal cells. **(A, B)** Barcode rank plots separately showing the detected knee and inflection points for fibrotic and regenerative wound dermal cells. **(C, D)** The expression of all genes, ribosomal genes, and mitochondrial genes in each cell was shown for fibrotic and regenerative wound dermal cells. **(E, F)** The proportions of mitochondrial and ribosomal genes expressed in each cell were counted for fibrotic and regenerative wound dermal cells.

Supplementary Table 1 The list of novel marker genes identified in each cell type.

## Abbreviations

scRNA-seq: single-cell RNA sequencing; GEO: Gene Expression Omnibus; PCA: principal component analysis; UMAP: Uniform Manifold Approximation and Projection; FC: fold change; GO: Gene Ontology; KEGG: Kyoto Encyclopedia of Genes and Genomes; PPI: protein–protein interaction; STRING: Search Tool for the Retrieval of Interacting Genes; GSVA: Gene Set Variation Analysis; ssGSEA: single-sample gene set enrichment analysis.
