# Corrigendum: Single-cell RNA-seq analysis reveals cellular functional heterogeneity in dermis between fibrotic and regenerative wound healing fates

**DOI:** 10.3389/fimmu.2023.1175360

**Published:** 2023-03-31

**Authors:** Cao-Jie Chen, Hiroki Kajita, Kento Takaya, Noriko Aramaki-Hattori, Shigeki Sakai, Toru Asou, Kazuo Kishi

**Affiliations:** ^1^ Department of Plastic and Reconstructive Surgery, Keio University School of Medicine, Tokyo, Japan; ^2^ Department of Plastic Surgery, Tokyo Cosmetic Surgery Clinic, Tokyo, Japan

**Keywords:** skin wound healing, fibrosis, regeneration, myofibroblast, macrophage, single-cell RNA sequencing

In the published article, there was an error in **Figures 2C, D** and **F** as published. The dot size for EN1 gene in different cell types in **Figure 2D** was wrong because we mislabeled the gene name during the production of the picture. Due to the same reason, the **Figure 2F** was also wrongly placed. In addition, we want to replace **Figure 2C** to add more feature genes (top 15, previously was top 10) in the heatmap to better characterize cell-type-specific gene expression patterns. The corrected **Figures 2C, D** and **F** appear below.

**Figure 2:**
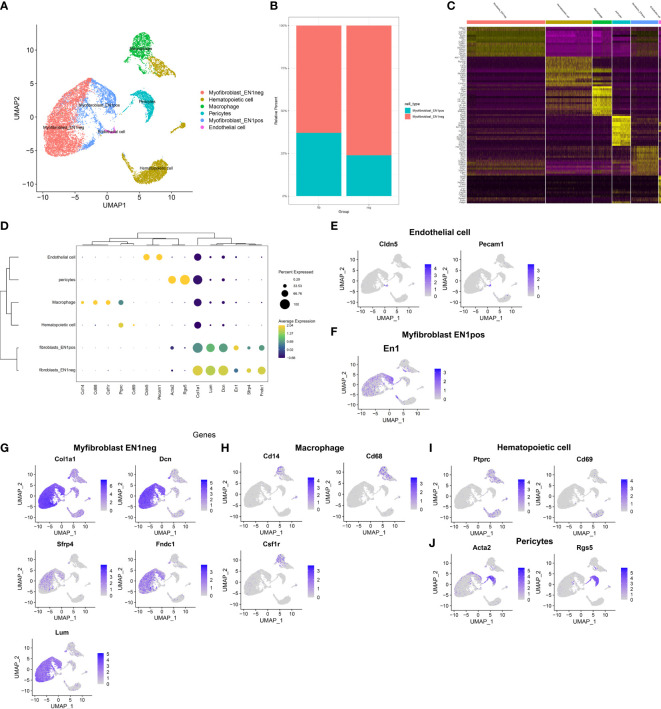
Identification of cell types and their marker genes across fibrotic and regenerative wound dermal cells. **(A)** UMAP plots showing cell types identified by marker genes. Each cell type was colored by a unique color. **(B)** The cell ratio of EN1-negative and -positive myofibroblasts among fibrotic and regenerative wound dermal cells. **(C)** Heatmap visualizing cell-type-specific gene expression patterns. Each column represented the average expression after cells were grouped. **(D)** Integrated analysis showing marker genes across cell types. The size of each circle reflected the percentage of cells in each cell type where the gene was detected, and the color shadow reflected the average expression level within each cell type. **(E–J)** UMAP plots of expression of the marker genes for endothelial cells, EN1-negative and -positive myofibroblasts, macrophages, hematopoietic cells, and pericytes.

The authors apologize for this error and state that this does not change the scientific conclusions of the article in any way. The original article has been updated.

