# Supplementary figures and images for: Single-Cell RNA-seq Analysis Reveals Cellular Functional Heterogeneity in Dermis Between Fibrotic and Regenerative Wound Healing Fates
Source: Front Immunol. 2022 May 17;13:875407. doi: 10.3389/fimmu.2022.875407 (PMC9156976; doi:10.3389/fimmu.2022.875407)

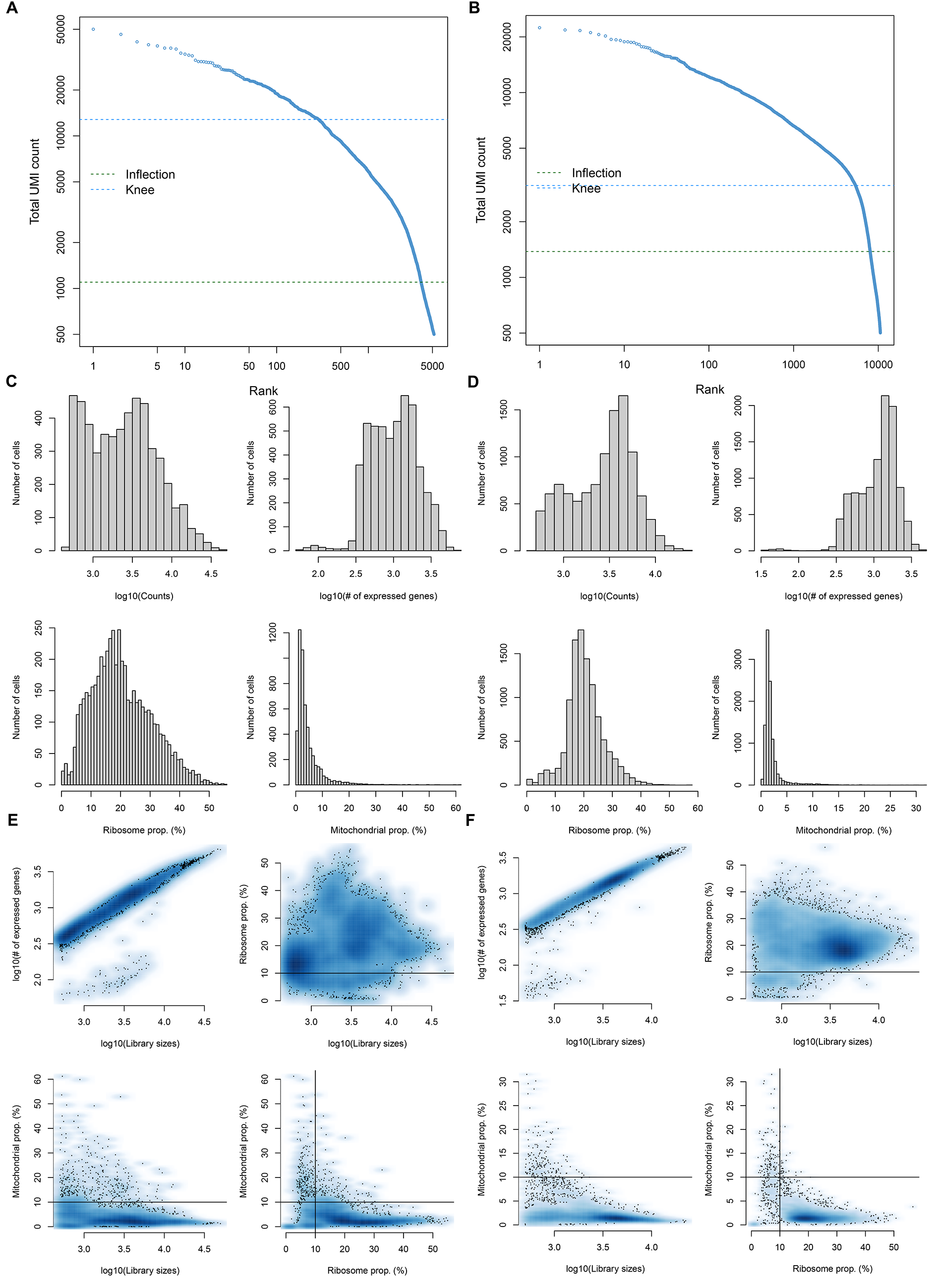

Supplement: Supplementary Figure 1 — Quality control of scRNA-seq data of fibrotic and regenerative wound dermal cells. (A, B) Barcode rank plots separately showing the detected knee and inflection points for fibrotic and regenerative wound dermal cells. (C, D) The expression of all genes, ribosomal genes, and mitochondrial genes in each cell was shown for fibrotic and regenerative wound dermal cells. (E, F) The proportions of mitochondrial and ribosomal genes expressed in each cell were counted for fibrotic and regenerative wound dermal cells. [file Image_1.tif]
